# Variation in preoperative self-administered Staphylococcus decolonization protocols for elective knee and hip arthroplasty across hospitals in Ontario, Canada: a cross-sectional study

**DOI:** 10.1017/ash.2025.10206

**Published:** 2025-12-09

**Authors:** Annie Liu, Ian Kudryk, Steve Mann, Yuka Asai, Anthony D Bai

**Affiliations:** 1 School of Medicine, Queen’s University, Kingston, ON, Canada; 2 Infection Prevention and Control, Kingston Health Sciences Centre, Kingston, ON, Canada; 3 Division of Orthopedic Surgery, Department of Surgery, Queen’s University, Kingston, ON, Canada; 4 Division of Dermatology, Department of Medicine, Queen’s University, Kingston, ON, Canada; 5 Division of Infectious Diseases, Department of Medicine, Queen’s University, Kingston, ON, Canada

## Abstract

In 2022, the Ontario Ministry of Health recommended preoperative nasal mupirocin and chlorhexidine body wash for *Staphylococcus aureus* decolonization. This 2025 cross-sectional study of 61 Ontario hospitals showed heterogeneity in decolonization protocols prior to hip and knee arthroplasty. Only 6.6% of hospitals indicated both recommended measures, highlighting an evidence-practice gap.

## Background

Surgical site infections (SSIs) and prosthetic joint infections (PJIs) following total knee or hip replacements are serious and frequent complications.^1–3^ *Staphylococcus aureus* is the most common pathogen, accounting for up to 33% of all PJIs.^1^ *S. aureus* decolonization using topical mupirocin ointment in the anterior nares and chlorhexidine gluconate body wash reduces the infection risk.^4,5^

In 2022, after a review of evidence indicating cost-effectiveness, the provincial government of Ontario published recommendations for universal decolonization using nasal mupirocin and chlorhexidine gluconate body wash prior to surgery.^6,7^ For context, Ontario is the most populous province in Canada with population of approximately 16 million. The healthcare is publicly funded and overseen by the Ministry of Health, part of the provincial government. Hip and knee arthroplasties are offered almost exclusively in publicly funded hospitals.

Now three years since the release of these guidelines, we conducted a cross-sectional study of hospitals in Ontario, Canada to characterize the variations in preoperative decolonization protocols intended for patient self-administration at home prior to elective total knee and hip arthroplasty.

## Methods

### Study design

This study was a cross-sectional study to evaluate the content and consistency of presurgical decolonization protocols provided to patients undergoing hip or knee arthroplasty across Ontario hospitals. Research ethics approval was not required as the study did not involve human participants or the collection of personal health information.

### Study sample

**Figure 1.**
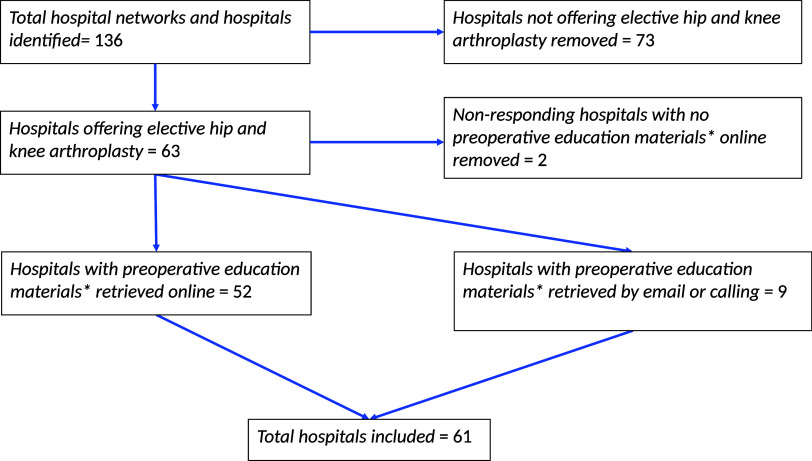
Hospital selection flow diagram. Preoperative education materials* comprise patient information pamphlets (knee and hip replacement, preparing for surgery, same day surgery), presurgical showering and chlorhexidine or other cleanser instructions, and surgical checklists.

From April 15 to July 12, 2025, we conducted a single-stage total population sampling approach to include all Ontario hospitals that offer routine elective total hip and knee arthroplasty surgery. Based on a complete list of hospitals from the Ontario government website,^8^ we included only hospitals that provide routine elective total hip and knee arthroplasty surgery based on the hospital website or direct communication with hospital staff (Figure 1).

### Data collection

For each hospital, we retrieved the most recent publicly available online preoperative education materials on bacterial decolonization and personal hygiene regimens for total hip and knee arthroplasty surgery. To supplement missing or unclear information, we contacted hospital administrators and surgical clinic staff via email and telephone (Figure 1).

All data were entered into a standardized information extraction sheet. Variables collected included hospital geographic region, format and subject of patient education materials, and details of the preoperative self-administered decolonization and hygiene protocols. Specifically, decolonization variables included screening procedures, type of decolonization (nasal and/or skin), application instructions, frequency of use, and anatomical site of application. Personal hygiene variables included recommendations for shaving, skin care, hair and nail care, appropriate clothing, bedding preparation, and the management of jewelry and piercings.

### Statistical analysis

We used counts and frequencies to describe the proportion of hospitals implementing different decolonization and hygiene regimens. Of the 63 eligible hospitals, only two (3.2%) hospitals did not have available instructions. Given the low nonavailability rate, complete case analysis was done.

## Results

This study included 61 hospitals across Ontario (Figure 1). The description of hospitals, decolonization regimens, and personal hygiene protocols are provided in Table 1.

**Table 1.** Preoperative *Staphylococcus aureus* decolonization regimens across hospitals

	Hospitals *N* = 61 (%)
**Hospital characteristics**	
Academic centers	13 (21.3%)
Community centers	48 (78.7%)
**Nasal decolonization**	
Nasal screening for *Staphylococcus aureus*	2 (3.3%)
Nasal mupirocin ointment	4 (6.6%)
**Type of body wash**	
Antiseptic wash (chlorhexidine)	38 (62.3%)
Antibacterial soap	4 (6.6%)
Soap unspecified	7 (11.5%)
Not specified	12 (19.7%)
**Frequency of body wash application**	
Once	18 (29.5%)
Twice	33 (54.1%)
Three or more times	2 (3.3%)
Not specified	8 (13.1%)
**Anatomical site for body wash application**	
Entire body from the neck down	19 (31.1%)
Unilateral limb	7 (11.5%)
Operative area only	2 (3.3%)
Mid-abdomen down	1 (1.6%)
Not specified	32 (52.5%)
**Decolonization regimen combinations**	
Both nasal mupirocin ointment and antiseptic skin cleansing (chlorhexidine)	4 (6.6%)
Antiseptic skin cleansing only (chlorhexidine)	34 (55.7%)
Did not describe any decolonization regimen involving antiseptic skin cleansing or nasal mupirocin ointment	23 (37.7%)
**Personal hygiene instructions**	
Do not shave	47 (77.0%)
Avoid lotions, creams, powders, oils, moisturizers, bath scents, aftershave	35 (57.4%)
No nail polish	44 (72.1%)
Remove all jewelry	48 (78.7%)

The most common decolonization approach was antiseptic skin cleansing without nasal mupirocin treatment in 34 (55.7%) hospitals. The most frequent skin wash regimen used by 33 (54.1%) hospitals involved showering twice, once the evening before, and once the morning of the surgery. The most common anatomical area to cleanse was the entire body from the neck down, specified by 19 (31.1%) hospitals. Chlorhexidine body wash and/or mupirocin topical treatment may be paid out-of-pocket by patients or provided for free by hospitals depending on the hospital site based on the online information or communication with hospital.

Only four (6.6%) hospitals advised both preoperative nasal mupirocin and chlorhexidine body wash decolonization, fully adhering to the 2022 Ontario Health recommendations. Of these four hospitals, two (3.3%) applied both measures universally, while the other two (3.3%) implemented chlorhexidine body wash universally and nasal mupirocin selectively based on *S. aureus* screening results.

Overall, most hospitals advised against shaving, recommended avoiding skin products postshower, and instructed removing nail polish and jewelry prior to surgery (Table 1).

## Discussion

Our cross-sectional study of 61 Ontario hospitals examined self-administered preoperative decolonization and hygiene protocols for patients undergoing elective total hip and knee arthroplasty. The findings revealed wide variation in the use of nasal mupirocin, chlorhexidine body wash, and other personal hygiene measures. Although three years have passed since Ontario Health’s recommendation to implement both decolonization interventions, only four (6.6%) hospitals have adopted both nasal mupirocin and chlorhexidine body wash.

Numerous studies have demonstrated that nasal mupirocin and chlorhexidine body wash decreased risk of SSIs.^4,6^ However, there is little data on the translation of these findings into real-world clinical practice. To our knowledge, this is the first comprehensive study describing institutional variations in the implementation of *S. aureus* decolonization regimens prior to elective hip and knee arthroplasty despite a published provincial guideline recommendation.

A key strength of our study was the high rate (96.8%) of available instructions that encompasses both academic and community hospitals of various sizes, making our findings generalizable. Moreover, the recent provincial government recommendation in 2022 allowed an opportunity to evaluate how closely hospitals adhere to evidence-based guidelines.

There are several limitations that warrant consideration. First, the date of publication of patient-directed materials ranged from 2013 to 2025 or lacked a version date, raising the possibility that certain instructions may be outdated. Additionally, publicly available protocols may not fully reflect real-world practice, as clinical implementation may vary depending on the surgeon, patient, and other factors. Further, comparisons between institutions may be limited by differences in document clarity or completeness rather than true variances in practice. Lastly, these reported directives do not capture patient-level adherences, which may influence protocol effectiveness. Nevertheless, this study is the most comprehensive and up-to-date overview of decolonization and personal hygiene practice patterns per hospital sites across Ontario, Canada.

Our study findings have significant implications for preoperative care in hospitals. First, the wide variation in decolonization practices should be addressed because most patients do not undergo both nasal and skin decolonization. Given the 2022 publication of provincial guidelines, hospitals should work toward harmonizing the preoperative regimens so that it is consistent and aligns with evidence-based recommendations. Furthermore, we observed considerable variability in the clarity and comprehensiveness of preoperative educational materials provided to patients. Elevating the quality and uniformity of these resources to ensure they are clear and thorough will support patient understanding and protocol adherence.

Variations in personal hygiene recommendations and antiseptic body wash regimens, such as application frequency and anatomical cleansing site, reflect the lack of evidence in the literature regarding the optimal decolonization approach.^9^ While most hospitals recommend self-administering antiseptic washes twice, some protocols instruct patients to apply it up to four times from the neck down prior to surgery. Future studies should compare different regimens to find the simplest and most effective strategy.

Our study highlights an evidence-practice gap in preoperative *S. aureus* decolonization practices prior to knee and hip arthroplasty across Ontario hospitals. Future studies may focus on knowledge translation and quality improvement to better understand and reduce the barriers to implementing preoperative decolonization regimens. These barriers may include cost, challenges with integration into institutional workflows, logistic challenges in dispensation, and inadequate patient and staff education or compliance.^6,10^
